# An Investigation of Cancer Rates in the Argentia Region, Newfoundland and Labrador: An Ecological Study

**DOI:** 10.1155/2015/421562

**Published:** 2015-11-08

**Authors:** Pauline Duke, Marshall Godwin, Mandy Peach, Jacqueline Fortier, Stephen Bornstein, Sharon Buehler, Farah McCrate, Andrea Pike, Peizhong Peter Wang, Richard M. Cullen

**Affiliations:** ^1^Primary Healthcare Research Unit, Memorial University, St. John's, NL, Canada A1B 3V6; ^2^Discipline of Family Medicine, Memorial University, St. John's, NL, Canada A1B 3V6; ^3^Newfoundland and Labrador Centre for Applied Health Research, St. John's, NL, Canada A1B 2X5; ^4^Community Health and Humanities, Memorial University, St. John's, NL, Canada A1B 3V6; ^5^Newfoundland and Labrador Cancer Registry, Eastern Health Authority, St. John's, NL, Canada A1B 3V6

## Abstract

*Background*. The Argentia region of Newfoundland and Labrador, Canada, was home to a US naval base during a 40-year period between the 1940s and the 1990s. Activities on the base resulted in contamination of the soil and groundwater in the region with chemicals such as heavy metals and dioxins, and residents have expressed concern about higher rates of cancer in their community. This study investigated the rate of cancer diagnosis that is disproportionately high in the Argentia region. *Methods*. Cases of cancer diagnosed between 1985 and 2011 were obtained for the Argentia region, two comparison communities, and the province of Newfoundland and Labrador. Crude and age-standardized incidence rates of cancer diagnosis were calculated and compared. The crude incidence rate was adjusted for differences in age demographics using census data, and age-standardized incidence rates were compared. *Results*. Although the Argentia region had a higher crude rate of cancer diagnosis, the age-standardized incidence rate did not differ significantly from the comparison communities or the provincial average. Argentia has an aging population, which may have influenced the perception of increased cancer diagnosis in the community. *Conclusions*. We did not detect an increased burden of cancer in the Argentia region.

## 1. Introduction

As part of a lend-lease agreement between the United States and Great Britain, land on the Argentia peninsula of what was then the Dominion of Newfoundland was leased to the United States government for construction of a navy base during WWII [1]. As one of the six facilities in British territories that were leased, the base at Argentia was of great strategic importance for shipping and defense [1, 2]. Newfoundland joined Canada in 1949 and the land occupied by the Argentia base, in Placentia Bay, is part of the province of Newfoundland and Labrador (NL), Canada, within the boundaries of the municipality of Placentia, NL. The base remained operational after the conclusion of the Second World War and throughout much of the Cold War period [2].

The base was the site of a series of chemical and biological aerial spray tests performed by the American military during the mid-1960s to evaluate the readiness of US military vessels for potential chemical and biological attacks [3]. As part of the testing, a combination of the biological agent* Bacillus globigii* and fluorescent zinc cadmium sulfide particles was sprayed from A-4 and C-47 aircraft, respectively [3, 4].

The United States Navy closed the base at Argentia in 1994 and site contamination was extensive [2]. Landfills in the areas contained lead paint, asbestos, polychlorinated biphenyls, dioxins and furans, heavy metals, petroleum hydrocarbons, and a variety of other wastes [2]. Environmental assessments of the area conducted in the mid-1990s found evidence of contamination in ground, soil, and pond water at sites across the base, including evidence of leaks from chemical storage tanks and pipelines [2]. Many contaminants, including heavy metals and dioxins, were present at levels considered to be a concern for human health [2]. Assessment and remediation of the site were undertaken by the Canadian government between 1996 and 2002 at a cost of well over $100 million dollars [5].

Concerns about the health implications of living in close proximity to potentially hazardous sites or in communities with exposure to industrial or agricultural chemicals are not uncommon [6, 7]. Epidemiological assessments are conducted in a hundred of communities every year in the United States alone [6], including communities near military installations such as Fort Detrick in Maryland [8], Northway airfield in Alaska [9], Fort Totten in New York [10], and a military antennae installation in Cyprus [11]. Community concerns may stem from confirmed exposure to environmental contamination [8–10], first- or second-hand occupational exposure among community members [12, 13], or perceived risks associated with these sites [11]. Unusual clusters of diseases that occur near industrial sites such as military bases and nuclear power plants, such as cancer, may raise concern for people living nearby and could warrant further investigation [8, 9, 12].

Some individuals living in the Argentia region have voiced the concern that the incidence of cancer in their communities is higher than that of the province of Newfoundland and Labrador as a whole. In the early 1990s an environmental health assessment was conducted in response to media reports of lymphopenia among residents of the Argentia region [14]. The assessment did not find statistically significant increases in the rate of miscarriages, stillbirths, or overall mortality. There was a significantly higher rate of brain and bladder cancer in the region, but the authors attributed that to random variability in a small sample. One of the recommendations of the report was the ongoing evaluation of the health of the population in the Argentia region.

Our objective is to examine the incident cancer rates in the Argentia region of Newfoundland compared to two control communities and determine whether the established presence of environmental contaminants in the past may be associated with a higher incidence of cancer in this region.

## 2. Materials and Methods

**Figure 1:**
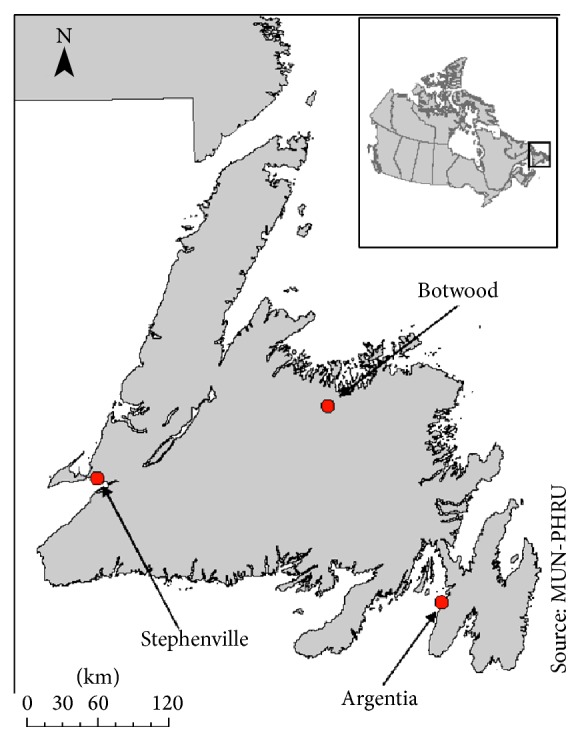
Map of the island of Newfoundland with the communities of Argentia, Botwood, and Stephenville indicated. Source: Province/Territories Boundary File, 2011 Census. Statistics Canada Catalogue number 92-160-X.

We selected the communities of Stephenville and Botwood to act as comparators to Argentia in our analysis of the rates of cancer diagnosis (Figure 1). The community of Stephenville was selected as it was also the site of a US military base from 1941 to 1966. The community of Botwood was selected as a control, as there was no historical military presence in the area.

Cancer data was obtained from the Newfoundland and Labrador Cancer Registry. Data were grouped into five 5-year increments (1985 to 2009) and one 2-year increment (2010-2011) for brain, breast, colorectal, lung, and prostate cancers. For cervical, gastric, kidney, liver, and ovarian cancers, the data were grouped into four 5-year increments (1985–2004) and one 6-year increment (2005–2011). In the latter instance, the final two time intervals were combined by the registry to suppress the relatively small numbers. For all types of cancer, the data were organized into four 5-year increments (1985–2004) and one 7-year increment (2005–2011). The crude diagnosis rate for cancer of the breast, brain, colon, lung, and prostate was calculated, as was the overall cancer diagnosis rate. Rates were calculated in all three communities as well as the province as a whole, and all rates are presented per 100,000 person-years.

Custom census data for the three communities was obtained from Statistics Canada for the years 1981–2011. The census data was used to calculate age-standardized incidence rates (ASIRs) with 95% confidence intervals for each community, using the NL provincial rate as a reference.

All methods were approved by the Health Research Ethics Authority of Newfoundland and Labrador.

## 3. Results and Discussion

**Figure 2:**
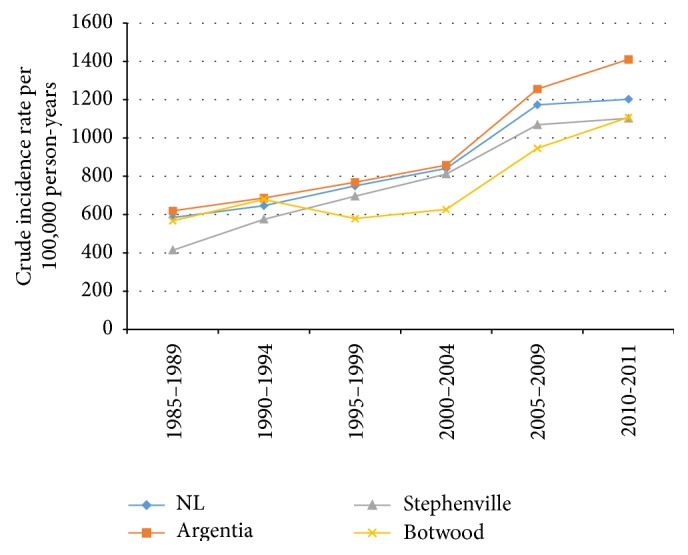
Crude incidence rates (per 100,000 person-years) for all types of cancer in the province of Newfoundland and Labrador and Argentia, Stephenville, and Botwood stratified by year period.

The crude incidence rate for cancer in the Argentia region was higher than the provincial crude rate for the periods of 1985–1989 (702.7 versus 648.4 cases per 100,000 person-years), 1990–1994 (739.9 versus 737.4 cases per 100,000 person-years), 2000–2004 (957.1 versus 936.3 cases per 100,000 person-years), and 2005–2011 (1278.7 versus 1189.3 cases per 100,000 person-years); the crude incidence rate for the Argentia region was lower than the provincial average for the time period 1995–1999 (853.7 versus 860.5 cases per 100,000 person-years) (Table 1). In the communities of Stephenville and Botwood the crude incidence rate was lower than the provincial average for all time periods except Botwood in 1990–1994 (759.2 versus 737.4 cases per 100,000 person-years) (Table 1, Figure 2).

**Figure 3:**
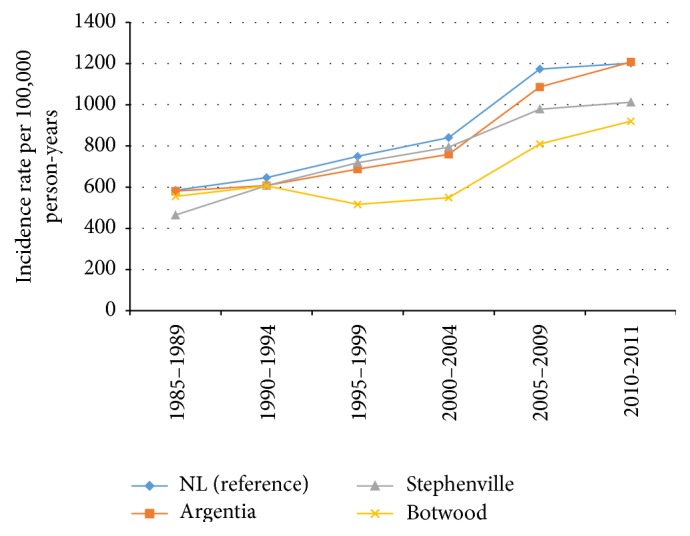
Age-standardized incidence rates (per 100,000 person-years) stratified by year for all types of cancer in Newfoundland and Labrador (reference) and Argentia, Stephenville, and Botwood.

After completing age-standardization of the incidence rate, the incidence rate in all communities was less than the provincial average, with the exception of Argentia in the time period 1985–1989 (658.7 versus 648.4 cases per 100,000 person-years) (Figure 3).

**Figure 4:**
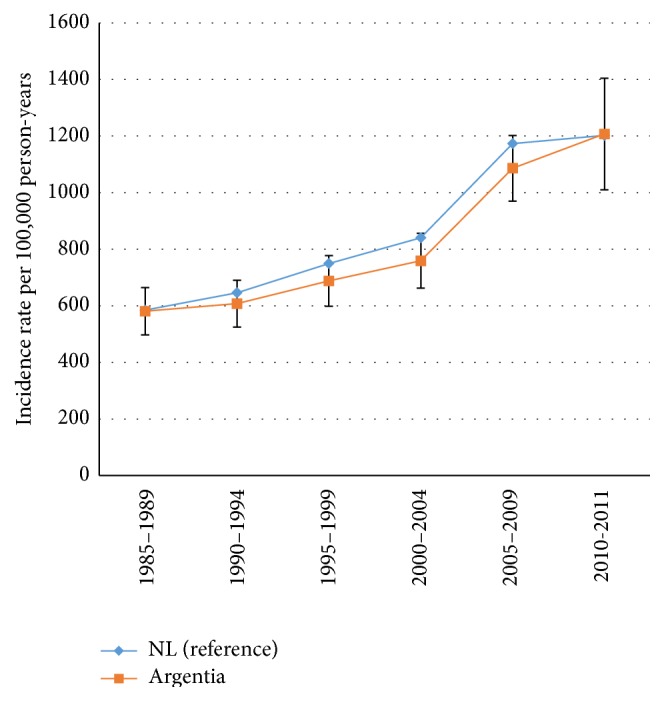
Age-standardized incidence rates (per 100,000 person-years) with 95% confidence limits for all types of cancer in Newfoundland and Labrador (reference) and Argentia region.

However, the rate is well within the ASIR 95% confidence interval (569.8–747.5 cases per 100,000 person-years) (Figure 4).

**Figure 5:**
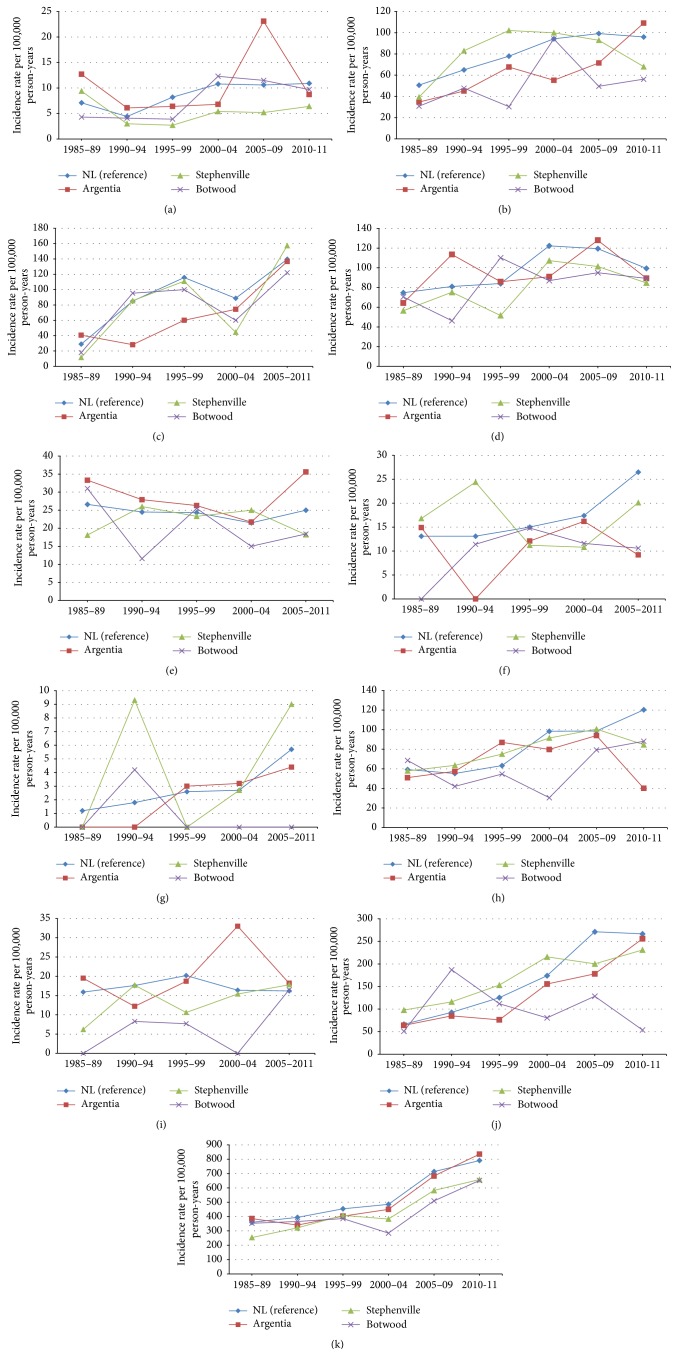
Age-standardized incidence rates (per 100,000 person-years) in the province of Newfoundland and Labrador and the Argentia, Stephenville, and Botwood regions stratified by year period, for (a) brain cancer, (b) breast cancer, (c) cervical cancer, (d) colorectal cancer, (e) gastric cancer, (f) kidney cancer, (g) liver cancer, (h) lung cancer, (i) ovarian cancer, (j) prostate cancer, and (k) all other nonpediatric, nonhematologic cancers.

ASIRs were calculated for specific types of cancer, including brain, breast, cervical, colorectal, gastric, kidney, liver, lung, ovarian, and prostate cancers, as well as for other types of cancer (Figures 5(a)–5(k); Table 2). As with the overall incidence rate, we observed no significant difference in incidence rates when standardized by age with the exception of gastric cancer, which was higher for four of the five time intervals (Figure 5(e)).

The Atlantic region of Canada, including the province of NL, is known to be among those having the highest incidence rates of cancer in the country [15], as well as an aging population [16]. These factors are intertwined and likely played a role in the perception by people living in Argentia that their burden of cancer was higher. People are more likely to be diagnosed with cancer as they get older [15], and Argentia's population is aging; 10.78% of the population was over the age of 65 in 1985, but that percentage had grown to 18.06% by 2011.

The general trend of increased cancer incidence in all three communities and the province as a whole (Figure 2) is likely due to a combination of the increasing age of the population and improved case ascertainment rates at the cancer registry, which will be discussed further as a limitation of this study. We can find no explanation for the comparatively low cancer rate in Botwood, our control community.

The incidence rates for individual types of cancer must be interpreted with caution due to the relatively small number of cases. Among the individual types of cancer we examined, the vast majority were diagnosed in fewer than 50 people per community in each time period, and such small numbers are sensitive to chance variation and any rates calculated from such small numbers have large confidence intervals. For example, there was a sharp increase in the diagnosis of brain cancer in the Argentia region in the period 2005–2009 from approximately 7 to 23 cases per 100,000 person-years (Figure 5(a)), but the incidence rate returned to normal levels in the next time interval. Another example is gastric cancer, for which the ASIR in the Argentia region was higher than the provincial rate in each time interval, but not at a rate that reached statistical significance (Figure 5(k)). It should be noted that the southeast coast of Newfoundland and Labrador, which includes the community of Argentia, is known to have a number of families with hereditary diffuse gastric cancer [17], and we suggest that the modest increased rate of gastric cancer may be related to this syndrome.

This study has some important limitations. The cancer registry in NL does not include either pediatric or hematologic cancers, and so we are unable to examine any potential trends in that data. Additionally, the cancer registry data was not, historically, as reliable as it is today. Incidence data in the registry has improved steadily over time, particularly following electronic linkage to provincial pathology laboratories.

The width of the confidence intervals around our rates and the previously mentioned fluctuations between time intervals demonstrate the relatively small communities we investigated. Although there was some fluctuation over time, the populations in these communities ranged from 10,000 to 12,000 in Argentia, 8,800 to 9,400 in Botwood, and 13,000 to 15,000 in Stephenville. We presented all data as rates and adjusted for age distributions, but these are all relatively small communities that are vulnerable to the statistically random fluctuations in disease incidence. This was noted in Buehler et al.'s study in the early 1990s [14] and remains a significant factor in our analysis.

Economically driven outmigration from the area further complicates the assessment of the impact of environmental contamination in the Argentia area. Between 1975 and 1993 the unemployment rate for the Argentia region was never below 10%, with a peak of 18% unemployment in 1984 [18]. Many individuals and families left the area to pursue economic opportunities in other parts of the province or in other provinces in Canada [19]. The rate of cancer diagnosis in this study is limited to those individuals diagnosed with cancer who were still living in the Argentia region at the time of their diagnosis; individuals who left the area and were subsequently diagnosed with cancer would not be captured in these rates. Attempting to truly quantify the cancer burden of those exposed to environmental contaminants within the Argentia region but who no longer reside there would be a time- and resource-intensive project.

Although we found no significant evidence of an increased risk of cancer in the Argentia region, the importance of investigating community concerns about a perceived increase in disease burden should not be underestimated. Cluster investigation guidelines published by the US Centers for Disease Control dating back to the early 1990s note that “the perception of a cluster in a community may be as important as, or more important than, an actual cluster” [8]. Concerns raised by people who feel they are at increased risk of developing certain disease due to environmental exposures provide an opportunity for epidemiological researchers to engage with communities, address their concerns using rigorous research methods, and provide answers based upon the available evidence.

Simpson et al. note that the results may run contrary to the expectation of people living in “what they believe is a cancer cluster” and underscore the importance of unbiased investigation, open lines of communication, and sensitivity to the concerns of the community [8].

## 4. Conclusions

Our results are similar to the results of Buehler et al.'s Review of the Health of the Population: Placentia/Long Harbour Area. We did not observe a significant increase in the cancer burden in the region after accounting for age. Cancer rates in the Argentia region of Newfoundland and Labrador are not higher than the rates in other communities or the province as a whole. The perception of a higher than average risk of cancer in the region may be due, in part, to the age of the population, as crude cancer diagnosis rates in Argentia are higher than comparison communities. Outmigration from the area complicated the assessment of the impact of environmental contamination in the Argentia area. Cancer diagnosis in this study is limited to those individuals diagnosed with cancer who were still living in the Argentia region at the time of their diagnosis; individuals who left the area and were subsequently diagnosed with cancer would not be captured in these rates.

## Figures and Tables

**Table 1:** Age-standardized incidence rates (ASIRs) per 100,000 person-years for all types of cancers in Argentia, Stephenville, and Botwood regions stratified by year period.

	1985–89	1990–94	1995–99	2000–04	2005–11
NL province (reference)					
Crude rate	648.4	737.4	860.5	936.3	1189.3
Argentia					
Crude rate	702.7	*739.9*	853.7	*957.1*	1278.7
ASIR	658.7	659.8	773.4	855.6	1107.6
(95% CI)	(569.8–747.5)	(573.4–746.2)	(677.8–869.1)	(752.0–959.2)	(1008.7–1206.5)
Stephenville					
Crude rate	453.5	672.0	792.3	882.0	1094.1
ASIR	508.1	708.2	817.5	866.5	1001.5
(95% CI)	(426.8–589.4)	(616.5–800.0)	(723.1–911.9)	(770.7–962.4)	(917.3–1085.7)
Botwood					
Crude rate	608.4	759.2	824.5	685.2	987.0
ASIR	594.5	684.0	743.4	607.0	844.3
(95% CI)	(493.9–695.2)	(581.5–786.5)	(639.1–847.7)	(511.1–702.8)	(750.9–937.7)

**Table 2:** Age-standardized incidence rates per 100,000 person-years for all types of cancers in Newfoundland and Labrador (reference), Argentia, Stephenville, and Botwood stratified by year period.

Community	Type	Age-standardized incidence rate (per 100,000 person-years)						
		1985–89	1990–94	1995–99	2000–04	2005–09^*∗*^	2010-11^*∗*^	2005–11^*∗*^
NL (reference)	Brain	7.1	4.4	8.2	10.8	10.6	10.9	
Argentia		12.7	6.1	6.4	6.8	23.1	8.7	
Stephenville		9.4	3	2.7	5.4	5.2	6.4	
Botwood		4.3	4.1	3.9	12.3	11.5	9.7	

NL (reference)	Breast	50.6	65	77.9	94.2	99.2	96	
Argentia		34.7	45.1	67.6	55.2	71.5	109	
Stephenville		39.3	83	102.1	99.9	92.9	68	
Botwood		30.8	47.8	30.3	94.1	49.5	56.2	

NL (reference)	Cervical	29	85	115.7	88.7			139.6
Argentia		40.7	28.2	60.1	74.3			136.7
Stephenville		11.6	85.6	110.9	44.5			157.3
Botwood		17.9	95.4	100	60.1			122.2

NL (reference)	Colorectal	74.7	81	84	122.3	119.4	99.4	
Argentia		64.3	113.5	85.9	91	128	89.4	
Stephenville		56.4	75.2	51.6	107.3	101.3	84.7	
Botwood		70.4	46.3	110.2	87	95	89.2	

NL (reference)	Gastric	26.6	24.5	24.3	21.5			25
Argentia		33.3	27.9	26.3	21.7			35.6
Stephenville		18.1	26	23.3	25			18.2
Botwood		31	11.6	25.5	15			18.4

NL (reference)	Kidney	13.1	13.1	15	17.4			26.5
Argentia		14.9	0	12.1	16.2			9.2
Stephenville		16.8	24.4	11.2	10.8			20.1
Botwood		0	11.4	14.8	11.6			10.6

NL (reference)	Liver	1.2	1.8	2.6	2.7			5.7
Argentia		0	0	3	3.2			4.4
Stephenville		0	9.3	0	2.7			9
Botwood		0	4.2	0	0			0

NL (reference)	Lung	59.3	55.3	63.3	98.4	98.7	120.3	
Argentia		51	57.4	87	79.8	94	40.1	
Stephenville		57.9	63.6	75.1	91.5	100.6	84.6	
Botwood		68.6	42.1	54.7	30.5	79.3	88.2	

NL (reference)	Ovarian	15.9	17.6	20.2	16.4			16.2
Argentia		19.5	12.2	18.7	33			18.2
Stephenville		6.2	17.7	10.6	15.4			17.8
Botwood		0	8.3	7.7	0			16.5

NL (reference)	Prostate	66.4	92.8	125.3	174	271.4	266.7	
Argentia		64.1	84.9	76.2	155.7	178.1	256	
Stephenville		97.8	116.1	153.2	215.8	200.2	231.1	
Botwood		51	187	111.9	80.6	128.5	53.7	

NL (reference)	Other	360.3	394.7	454.6	485.3	713.3		
Argentia		385.8	340.4	403	450.5	682.8		
Stephenville		253.8	321.9	408.2	383	582		
Botwood		353.5	365.9	386.1	284.6	509.1		

^*∗*^Note: for certain types of cancer the year periods 2005–2009 and 2010-2011 have been combined to suppress small numbers.

## Abbreviations

WWII: World War II
ASIR: Age-standardized incidence ratio
NL: Newfoundland and Labrador.
